# Ursolic Acid and Related Analogues: Triterpenoids with Broad Health Benefits

**DOI:** 10.3390/antiox10081161

**Published:** 2021-07-21

**Authors:** Huynh Nga Nguyen, Sarah L. Ullevig, John D. Short, Luxi Wang, Yong Joo Ahn, Reto Asmis

**Affiliations:** 1 Ardelyx Inc., Fremont, CA 94555, USA; nga.nancy.nguyen@gmail.com; 2 College for Health, Community and Policy, University of Texas, San Antonio, TX 78207, USA; sarah.ullevig@utsa.edu; 3 Department of Pharmacology, University of Texas Health Science Center, San Antonio, TX 78229-3900, USA; johnny.d.short@gmail.com; 4 Department of Internal Medicine, Wake Forest School of Medicine, Winston-Salem, NC 27157, USA; luxiwang1@gmail.com (L.W.); yojoahn@gmail.com (Y.J.A.)

**Keywords:** botanicals, nutraceutical, antioxidants, inflammation, metabolic diseases, atherosclerosis, cancer

## Abstract

Ursolic acid (UA) is a well-studied natural pentacyclic triterpenoid found in herbs, fruit and a number of traditional Chinese medicinal plants. UA has a broad range of biological activities and numerous potential health benefits. In this review, we summarize the current data on the bioavailability and pharmacokinetics of UA and review the literature on the biological activities of UA and its closest analogues in the context of inflammation, metabolic diseases, including liver and kidney diseases, obesity and diabetes, cardiovascular diseases, cancer, and neurological disorders. We end with a brief overview of UA’s main analogues with a special focus on a newly discovered naturally occurring analogue with intriguing biological properties and potential health benefits, 23-hydroxy ursolic acid.

## 1. Introduction

There is substantial evidence supporting the role of phytochemicals, which are categorized as secondary plant metabolites, as major contributors to the health benefits of diets rich in fruits and vegetables [1]. In contrast to primary metabolites, which are essential nutrients needed for fundamental metabolic processes that comprise over 99% of all metabolites in the plant, secondary metabolites are low in abundance and considered non-essential. However, phytochemicals are used for adaptive defense systems against environmental stresses [2,3], thereby providing an ecological advantage to optimize plant interactions with other plants, microbes, and animals. Phytochemicals encompass a wide range of compounds and are classified into three primary groups based on their biochemical origin and structural similarities: polyphenols, alkaloids, and terpenoids [2]. Terpenoids are the largest group of phytochemicals and are comprised of both primary and secondary metabolites [2].

Terpenoids are derived from one or more 5-carbon isoprenoid units and can be further characterized by the number of isoprene units and carbon atoms (C10, C15, C20, C30, C40) [2]. Hemiterpenoids (C5) and sesterpenoids (C25) are rarely found in nature [2]. Isoprenoids are synthesized in one of two ways: via the mevalonic acid (MVA) pathway that originates from two acetyl-CoA molecules or the methylerythritol 4-phosphate (MEP) pathway that originates from pyruvate and D-glyceraldehyde 3 phosphate [4]. The MVA pathway is utilized in plants, algae, bacteria, and mammals for steroid hormone and cholesterol synthesis. The MEP pathway, located in the plastid of the plant cell, is only found in plants, algae, and some bacteria and forms mono-, di- and tetraterpenes [4] (MVA and MEP pathways are reviewed here [5,6,7,8,9].

**Figure 1:**
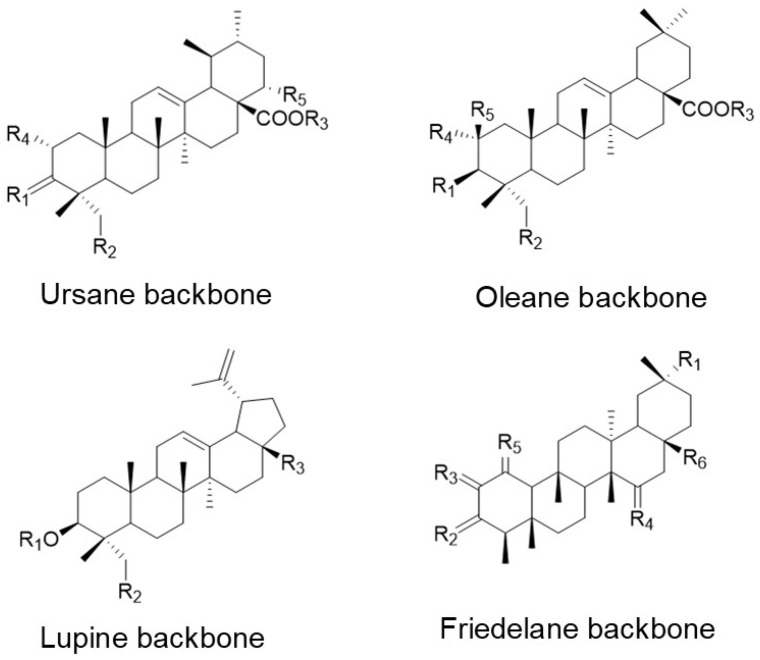
Four basic ring skeletons of pentacyclic triterpenoids.

**Figure 2:**
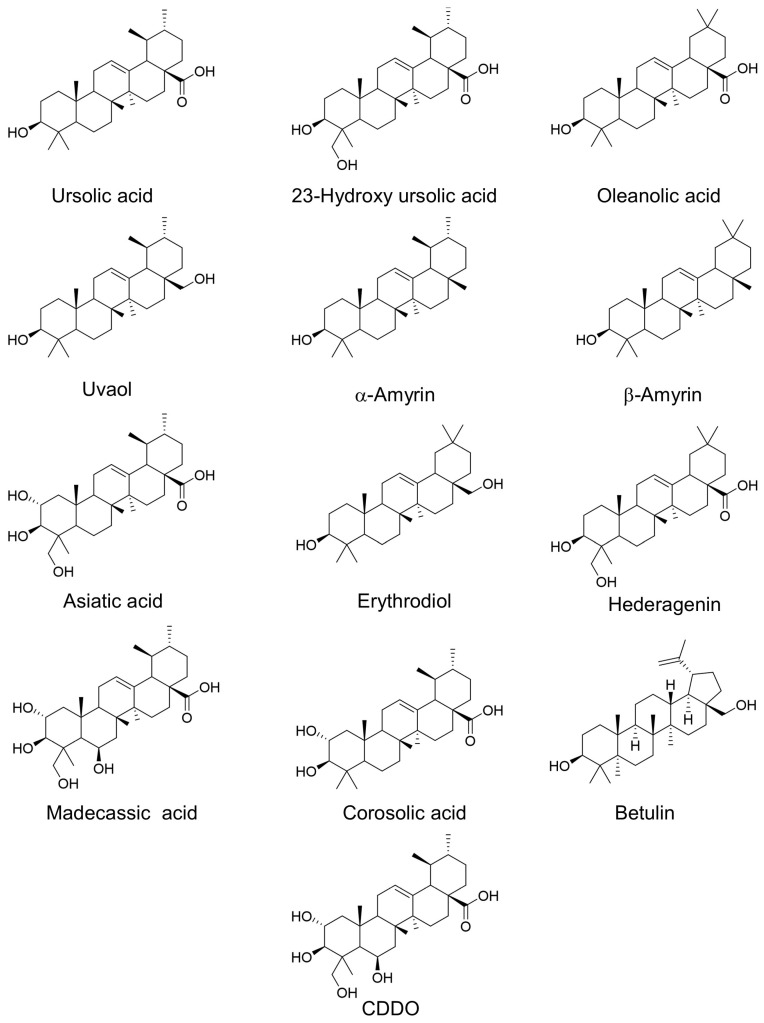
Structures of ursolic acid and key related pentacyclic triterpenoids.

Triterpenoids are composed of six isoprene units formed via the MVA pathway and are in plant resin, cork, waxy coatings and frequently associated with polysaccharide gums [10]. Triterpenoids occur naturally either unmodified or modified by glycosylation, which are referred to as saponins. Non-glycosylated triterpenoids provide a protective waterproofing layer found in plant cuticles, which provides a lipophilic membrane on leaves, stems, and fruits [10]. Pentacyclic triterpenoids are the most common and widely distributed triterpenoids and are intermediates in steroid hormone biosynthesis in both plants and mammals [2]. Pentacyclic triterpenoids are categorized by four basic ring skeletons: ursane (five six-member rings with one methyl at position 19 and 20), oleanane (five six-member rings with two methyl groups at position 20), friedelane (five six-member rings with methyl groups at C4, 5, 10, 8, 13), and lupine (four six-member rings and one five-member ring) [10] (Figure 1). Five of the most studied terpenoids are ursolic acid (UA) and asiatic acid (AA, ursane group), oleanolic acid (OA) and β-amyrin (oleanane group) and betulin (lupine group) (Figure 2).

Ursolic acid (UA) is a pentacyclic triterpenoid primarily synthesized through the MVA pathway, similar to steroid hormones in plants and mammals [4]. UA is found in the protective, waxy coating of apples and other fruits [11,12,13]. This review focuses on UA and related analogues in the ursane and oleanane groups, as numerous studies suggest these anti-inflammatory compounds may have significant health benefits and protect against various diseases, including metabolic disorders and obesity, cardiovascular diseases, cancer, and neurological disorders.

## 2. Bioavailability and Pharmacokinetics

The bioavailability and pharmacokinetics of UA have been characterized in both rodents and humans. In the Biopharmaceutics Classification System (BCS), UA is considered a class IV compound, exhibiting poor oral bioavailability, low solubility, and intestinal permeability, yet UA demonstrates surprisingly strong pharmacodynamic properties and bioactivities [14]. Several groups have quantified UA. Chen et al. measured plasma concentrations and tissue distributions of UA using liquid chromatography-mass spectrometry (LC-MS) in Sprague-Dawley rats given an oral dose of UA and showed that UA plasma levels peaked at 1.1 ± 0.31 μg/mL approximately 30 min after exposure [15]. The highest concentration of UA was found in the lung (1.5 μM) with decreasing concentrations found in the spleen, liver, cerebrum, heart and kidney [15]. Similarly, Liao et al. measured UA in plasma from rats fed Lu-Ying extract (80 mg UA/kg) and reported that plasma UA peaked at 0.65 µM UA 1 h after administration and showed a half-life of 4.3 h [16]. Similar concentrations of UA have been observed in mice, albeit with much slower kinetics. For example, C57BL6 mice fed a diet supplemented with 0.5% UA showed no detectable levels of UA after four weeks on the dietary regimen; but plasma levels reached 1.3 μM after 8 weeks [13]. In addition, UA as well as related triterpenes were found to remain intact in tissues, with the highest concentration found in the liver [13]. These data suggest that the biological effects of UA in vivo are a result of unmodified UA. In humans, plasma levels of UA have primarily been assessed with liquid chromatography-tandem mass spectrometry (LC/MS/MS). Hirsch et al. recently showed that a single oral dose of UA had very low bioavailability [17]. Using a 100 mg dose, this group also showed that only 4 of 14 subjects had detectable levels of UA. However, 9 of 14 subjects had detectable UA levels when dosed at 1000 mg. These authors suggested that poor absorption and rapid clearance may contribute to low levels of UA detected in these subjects. In support of this hypothesis, Zhu and colleagues have shown that intravenous infusion of healthy volunteers with UA in nanoliposomes at 98 mg/m^2^ resulted in plasma UA concentrations that peaked at 7.5 μM after 4 h [18].

In addition to the pharmacokinetics of UA, several groups have studied the compounds’ toxicity as well as that of many of its naturally occurring analogues. Our group demonstrated in human THP-1 cells that at concentrations below 30 μM, UA and ten of its naturally occurring analogues did not exhibit any significant toxicity [19]. Using a brine shrimp bioassay, Somova et al. showed that UA had no toxic effects on mice when they administered UA for 5 days at 60 mg/kg of body weight [20]. A recent repeated-dose toxicity study evaluated the long-term toxic effect of UA on clinical chemistry, hematology, coagulation, pathology/morphology, behavior, and motor skills in male and female Han-Wistar rats [21]. The animals received daily doses of 1000 mg/kg/day via oral gavage for 90 days. The solution was administered to both male and female Han-Wistar rats for 90 consecutive days. The authors found that this regime does not lead to toxic effects at any of the doses tested and they concluded that the no-observed-adverse-effect-level (NOAEL) for UA is likely to be higher than 1000 mg/kg/day. A clinical pharmacokinetic and safety study in healthy adult volunteers of UA at single oral doses up to 1000 mg also found no serious adverse event [17]. However, the authors observed low and variable bioavailability, which they attributed to low intestinal absorption due to poor water solubility, rapid elimination, and/or metabolism by the gut wall and liver. In addition to the numerous rodent studies that have found a wide range of beneficial effects of UA, even at high doses, (Table 1), these findings suggest that UA has low toxicity in both rodents and humans. Nevertheless, despite the UA’s low bioavailability, it is possible that UA is stored and accumulates in tissues, including brain, liver, kidney, heart, lung, bladder, colon, and the spleen. Possible long-term toxic effects in vivo have not been investigated and require further studies.

## 3. Inflammation

Inflammatory diseases have become major targets for drug development because their effects are wide and debilitating [22]. Major molecular targets include pro-inflammatory cytokines and their receptors, tumor necrosis factor (TNF-α), interleukin (IL)-1β, IL-2, interferon (IFN)-γ, NF-κB, mitogen-activated protein kinases (MAPK), and c-Jun-N-terminal kinases (JNK) [23]. Many plants in traditional medicine have been used to treat inflammation, but in many cases, their potent bioactive constituents are still being investigated. UA is credited with giving Calluna vulgaris or common heather, a plant used for treating inflammatory conditions, its anti-inflammatory properties [24]. These authors reported that treating rat macrophages with 1 μM UA decreased lipoxygenase product formation and cyclooxygenase activity. Using UA and OA as building blocks, the group of Michael Sporn synthesized 60 triterpenoids as anti-inflammatory agents [25]. Many of their synthetic compounds were potent inhibitors of LPS-induced COX-2 and iNOS expression in mouse macrophages [26].

COX-2 is one of the many genes regulated by NF-κB, a family of transcription factors that regulates the expression of genes involved in tumorigenesis, adhesion molecules, chemokines, proinflammatory cytokines, and cell cycle genes [27]. NF-κB is negatively regulated by the IκB proteins. Once phosphorylated, IκB is ubiquitinated and degraded by the proteasome, releasing NF-κB to freely translocate to the nucleus. UA’s beneficial effects have been linked to its ability to suppress genes associated with NF-κB activation. Shishioda et al. found that UA suppressed NF-κB activation by inhibiting IκB kinase and p65 phosphorylation in various tumorigenic cell lines, including Jurkat, HEK293, KBM-5, H1299, and U937 [27]. NF-κB also regulates lipoxygenase, MMP-9, and iNOS [28], which may explain the inhibitory effects of UA on iNOS expression [26]. UA also inhibits NF-κB, activation in human intestinal epithelial cells and macrophages [29].

Checker et al. showed that UA’s effects on NF-κB, AP-1, and NF-AT are at least partly responsible for its potent anti-inflammatory effects in mouse lymphocytes [30]. They showed that UA addition to mouse splenic lymphocytes inhibits lymphocyte proliferation in a dose-dependent manner, with maximal inhibition at 5 μM of UA. UA also inhibits CD4+ and CD8+ T- and B-cell proliferation. The authors went on to show that UA inhibits cytokine secretion by lymphocytes induced by Con A or anti-CD3/CD28 monoclonal antibody addition. Treatment of Con A-stimulated lymphocytes with 5 μM UA completely inhibits the secretion of IL-2, IL-4, IL-6, and IFNγ and suppressed MAPK, NF-κB, NF-AT, and AP-1 activation.

Interestingly, UA appears to be an inhibitor of human neutrophil elastase (HNE), an enzyme that regulates local inflammatory processes [31]. Feng et al. used an in vitro HNE inhibition assay and a mouse model of smoke-induced lung inflammation to test multiple pentacyclic triterpenoids and found that UA was the most potent compound (IC_50_ = 5.5 μM) [31].

Finally, we would like to mention an important caveat in working with UA initially reported by Ikeda et al. [32]. These authors found that aggregated UA—in their hands UA aggregated in culture medium—enhances the release of IL-1β in cultured mouse peritoneal macrophages. Pretreating cells with an anti-CD36 antibody reduces IL-1β release, suggesting that aggregated UA interacts with the CD36 receptor, a scavenger receptor that mediates the phagocytosis of apoptotic cells [33], and oxidized LDL, a mechanism that protects macrophages from the cytotoxicity of OxLDL [34]. Interestingly, Ikeda and colleagues also reported that intra-peritoneal injections of UA (50 mg/kg solubilized in corn oil repeated for 8 days) increased IL-1β release as well [32], suggesting that the high concentration of UA in the corn oil may have led to aggregate formation. This potential artifact may explain the few reports of pro-inflammatory activities of UA as reviewed by Ikeda and colleagues [4].

## 4. Metabolic Diseases

Studies highlighting UA’s anti-inflammatory and antioxidant properties have spurred research focused on utilizing UA to either treat or prevent various metabolic diseases, including obesity, hypertension, diabetes, cardiovascular disease, and liver and kidney diseases, which are discussed below.

### 4.1. Liver Disease

UA has hepatoprotective properties that were first discovered in the mid to late 1980s using traditional Chinese medicine preparations to protect against carbon tetrachloride (CCl_4_)-induced liver injury [35]. Many triterpenoid compounds similar in structure to UA show this same liver protection in mice [36]. Using CCL4-treated mice, UA prevented liver damage and protected against oxidative stress and inflammation by decreasing the activation of MAPK pathways, including JNK, p38 MAPK, and ERK and NF-κB [37]. In addition, using a rodent model of chronic ethanol-induced liver damage, both UA isolated from Eucalyptus tereticornis [38] or pure UA (10, 20, or 40 mg/kg/day) improved liver function as measured by aspartate aminotransferase (AST) and alanine aminotransferase (ALT) plasma concentrations, and increased circulating antioxidant plasma levels (glutathione, α-tocopherol, and ascorbic acid) [39]. Furthermore, UA protects the liver from HFD-induced hepatic steatosis [40,41,42,43]. The mechanisms underlying UA’s hepatic protective properties are still unclear, but possible mechanisms have been proposed, including inhibition of cytochrome P450 (CP450) [35,44], the induction of apoptosis in liver-damaging hepatic stellate cells [45], reduction of oxidative stress through activation of LKB1-AMPK signaling [46], activation of proliferator-activated receptor alpha (PPARα) to regulate lipid metabolism [47], and reduced inflammatory cytokine production in response to IL-6 [48].

### 4.2. Obesity and Diabetes

One of the first studies conducted with UA in regards to metabolic diseases found that after 6 weeks of intraperitoneal (i.p.) injections with UA, Dahl salt-sensitive rats showed reduced hypertension, lower blood glucose and total cholesterol levels, and increased expression of two key antioxidant enzymes, glutathione peroxidase (GPx), and superoxide dismutase (SOD) in of UA [20]. In subsequent studies conducted utilizing a mouse model of diet-induced obesity (C57BL/6 mice fed a HFD) and a mouse model of streptozotocin (STZ)-induced hyperglycemia to mimic diabetes, UA supplemented in a HFD or the drinking water or administered i.p., has consistently shown benefits, including a reduction in fat mass, increased skeletal muscle mass, improved glucose control, and reduced plasma lipid levels [40,41,42,49,50,51,52] (see Table 1 for details). In addition to UA’s ability to preserve antioxidant enzyme activity [20], an additional mechanism underlying the improved metabolic profile observed in animal studies may include UA’s ability to modulate adipogenesis and lipolysis. UA was found to attenuate adipogenesis via the LKB1/AMPK pathway [53] and stimulate lipolysis by upregulation of adipose triglyceride lipase in primary rat adipocytes [54], indicating another potential anti-obesity mechanism for UA. UA has also been reported to modulate mTORC1 signaling in muscle, although the directionality of that effect appears to be context-dependent. In C2C13 myotubules, UA inhibited the activation of mTOR by leucine [55] through suppression of mTOR lysosomal localization. On the other hand, UA administered to exercised Sprague-Dawley rats, was able to sustain exercise-induced mTORC1 activity [56].

As mentioned above, UA also lowers blood glucose levels. One mechanism through which UA reduces blood glucose levels is via the inhibition of protein tyrosine phosphatase 1B, an important phosphatase inhibitor of insulin-mediated signaling [68]. Furthermore, UA appears to help preserve pancreatic islet cells function as the compound protected pancreatic islet cells from STZ-induced damage and impaired insulin secretion [61]. UA’s ability to reduce blood glucose levels has led to a series of studies investigating the therapeutic potential of combining UA with established anti-diabetic drugs. For example, UA treatment combined with rosiglitazone in HFD-fed C57BL/6J reduces whole body weight gain, prevents hepatic lipid accumulation, decreased systolic and diastolic blood pressure, improved lipid status and lowered blood glucose levels more effectively than either compound alone [62,63].

### 4.3. Cardiovascular Disease

Recent studies, including two from our group, have reported cardioprotective properties of UA. UA supplemented in a HFD strongly suppressed atherosclerotic plaque formation and increased survival in a mouse model of diabetes-accelerated atherosclerosis [60]. UA-treated mice also showed reduced monocyte migration and recruitment of monocyte-derived macrophages in vivo, as well as reduced accumulation of inflammatory blood monocytes. Of note, in this study, UA was more potent than resveratrol in preventing atherosclerosis. We confirmed the atheroprotective properties of dietary UA in a classic mouse model of atherosclerosis, HFD-fed LDL receptor-deficient mice [19]. Both studies provide compelling evidence that UA’s anti-atherogenic activity is to a large extent mediated by the compound’s protective effects on blood monocytes, preventing nutrient stress-induced hyperreactivity to chemoattractants and the overrecruiting of monocyte-derived macrophages into tissues. The underlying molecular mechanism appears to involve inhibition of metabolic stress-induced Nox4 protein expression and increased protein S-glutathionylation, a marker of oxidative stress and redox signaling [33,60,78,79].

In apparent contradiction to our reports, Messner et al. found that UA supplementation in the drinking water induces endothelial cell apoptosis, inflammation, and increased atherosclerosis in APOE^−/−^ mice [80]. However, the UA concentration in the drinking water used in this study was 30 mM. These high concentrations are likely toxic to mice, causing tissue irritation and injury, which may account for the systemic inflammation and increased atherosclerosis in these mice reported by the authors [80]. In fact, a 24-h exposure of THP-1 monocytic cells to UA concentrations exceeding 10 µM, i.e., a 3000-fold lower concentration, is sufficient to promote cell death [19].

In addition to protecting against atherosclerosis, UA protects against isoproterenol-induced myocardial infarction (MI) in rats as evidenced by reduced enzyme markers of disease (creatine kinase-MB and lactate dehydrogenase), lipid biomarkers (LDL, TG, and FFA), DNA fragmentation through upregulation of anti-apoptotic protein (Bcl-2, Bcl-xl), downregulation of apoptotic proteins, including Bax, caspase-3, -8, and -9, cytochrome c, TNF-α, and FAS, and reduced oxidative stress in the plasma and heart tissue of these animals [57,58]. Oral UA also protects aortas of STZ-induced diabetic mice from vascular injury as indicated by reduced aortic damage and oxidative stress and a concomitant decrease in RAGE, p22, and NF-kB expression [59]. Taken together, these data suggest that dietary UA has potent cardio- and vasculoprotective and anti-atherogenic properties and may represent a new class of oral therapeutics for the prevention and treatment of cardiovascular diseases.

Both UA and its analog, OA, also have anti-hypertensive properties. When OA from Greek olive oil and Cape Town cultivar, or a 1:1 mixture of OA and UA extracted from African wild olive leaves was administered to Dahl salt-sensitive (DSS) rats, an insulin-resistant rat model of hypertension, these rats were protected from the development of severe hypertension and atherosclerosis [81]. In addition to the anti-hypertensive properties, daily application of the mixture for 6 weeks reduced heart rate, reduced hyperlipidemia, and exerted antioxidant and hypoglycemic properties in DSS rats [20]. Sundaresan et al. reported that orally administered UA alone significantly reduced blood pressure in HFD-fed C57BL/6J mice [62]. A single intragastric dose (50 mg/kg) of UA significantly reduced systolic and diastolic blood pressure without affecting the heart rate in male spontaneous hypertensive (SHR) Wistar rats [82]. In the same rat model (SHRs), oral administration of OA (1.08 mg/kg) for 4 weeks prevented elevated systolic and diastolic pressure [83]. This anti-hypertensive effect was mediated by the downregulation of secretory phospholipase A_2_ (sPLA_2_) and fatty acid synthase. Thus, in addition to their cardio-and vasculoprotective properties, both UA and OA also appear to exert anti-hypertensive effects.

### 4.4. Kidney Disease

Evidence from several studies suggests that UA protects against diabetic-induced kidney disease. UA supplemented in the diet (0.05, 0.1 or 0.2%) of STZ-induced diabetic mice preserved kidney function as measured by creatinine clearance, diminished flux through the renal polyol pathway, and decreased advanced glycation end products (AGEs) formation in urine [64,84]. In addition, 0.01% UA supplemented in the diet of STZ-induced diabetic mice decreased glomerular hypertrophy, collagen accumulation and suppressed the activation of inflammatory and oxidative pathways (STAT-3, ERK1/2 and JNK) and iNOS overexpression [64]. After 16 weeks on a diet supplemented with 0.02% UA, STZ-treated mice showed improved kidney function [66]. An animal model of carbon tetrachloride (CCl4)-induced kidney damage was utilized to investigate UA’s protective effects and found that UA prevents CCl4-induced nephrotoxicity, ROS, DNA damage, proinflammatory markers [85]. Furthermore, oral administration of UA (2, 5, 10 mg/kg) protected kidneys from gentamicin-induced damage in rats [67]. These findings strongly suggest that UA is a potential oral therapeutic or adjunct therapy for the treatment of kidney diseases.

In summary, oral administration of UA greatly improves health outcomes in a variety of rodent models of human metabolic diseases. A large body of data suggests that UA may be an effective oral therapy for both the preventive and treatment of metabolic disorders in humans as well as the chronic inflammatory diseases associated with these disorders. To date, only a single small clinical trial has been conducted to examine these potential health benefits in humans. In this randomized, double-blind, placebo-controlled clinical trial, 24 patients between 30 and 60 years of age, with a diagnosis of metabolic syndrome without treatment, were randomly assigned to two groups of 12 patients each, which either received orally 150 mg of UA or homologated placebo once a day for 12 weeks [86]. The authors report transient remission of metabolic syndrome, reducing body weight, BMI, waist circumference, and fasting glucose, as well as increasing insulin sensitivity in 50% of patients that received oral UA.

## 5. Cancer

UA’s anti-cancer properties have initially been described for the prevention of skin tumors [87], but more recently, UA has been studied in a wide variety of cancers, including bladder, colon, cervical, breast, liver, and lung (Table 2). Chronic inflammation and oxidative stress are intricately linked with cancer development, progression, and metastasis [88,89]. Many of the inflammatory pathways that are up-regulated in cancer cells are targets of UA and other triterpenoids [90]. UA mediates many of its anti-cancer effects through up-regulation of NF-ĸB [27,91,92], Bcl-2 [91], ICAM-1 [93], and PKC [94] and/or the downregulation of STAT3 [95], JNK [96], and p53 [91], resulting in apoptosis, reduced proliferation, and decreased angiogenesis thereby preventing cancer tumor formation and metastasis.

Apoptosis, i.e., programmed cell death, is triggered by intrinsic and extrinsic cellular pathways. At high doses, UA has been shown to activate the intrinsic pathway by inhibiting anti-apoptotic pathways such as NF-ĸB [27,91,92] and COX-2 [103,106], and FoxM1 [59], and up-regulation of pro-apoptotic pathways through the activation of caspases [98,99,100,101,102,103,104], JNK [101,102,117], p53 [91], and the Trail-mediated pathway [105,121].

Carcinogenesis is characterized by excessive cell proliferation. UA has been shown to inhibit cell proliferation by inhibiting MAPKs [109,119,120] or STAT3 activation pathways [95,109,115,116]. In human non-small cell lung cancer, UA also blocks cell cycle progression in a p53 and p21^WAF1^-dependent manner [117].

Metastasis is dependent on tumor angiogenesis and the regulation of proteases, peptidases, and adhesion molecules. UA and other triterpenoids reduce the angiogenic potential through down-regulation of hypoxia-inducible factor (HIF)-1α, vascular endothelial growth factor (VEGF), and IL-8 [93,118,122]. In addition, multiple studies in various cancer cell lines showed that UA down-regulates two gelatinases responsible for the breakdown of extracellular matrix involved in cancer metastasis, matrix metalloproteinase 9 (MMP-9), and MMP-2 [27,94,119,123]. Furthermore, UA treatment reduces the expression of the adhesion molecule, intercellular adhesion molecule-1 (ICAM-1) [93,119], which is another important regulator of cancer metastasis.

While many of UA’s anti-cancer effects have been reported in cell lines, UA shows similar potency in rodent tumor models. Topical application of UA extracted from rosemary reduced the number of tumors formed in a CD-I mouse model of skin tumor [87]. DMBA and 12-O-tetradecanoylphorbol-13-acetate (TPA)-induced skin tumors were treated with rosemary extract (3.6 mg in 299 μL acetone) for 19 weeks, reducing tumor formation by 99%. This effect was attributed to UA and carnosol. Methanol-extracted UA at 0.2, 0.6, and 2.0 μM doses was found to significantly reduce tumors. At a dose of 2.0 μM, UA reduced tumor formation by 45%, whereas lower doses reduced tumor formation by 5–20% [54]. In SENCAR mice, topical application of UA, but not resveratrol, also reduced skin cancer induced by DMBA and TPA treatment. UA treatment also reduced COX2 and IL-6 mRNA expression in tumor-induced mice [124].

Dietary studies show that even low doses of UA are effective against cancer. In a mouse breast cancer model, mice on a 3-week diet of UA (0.05%, 0.1%, and 0.25% *w*/*w*) demonstrated reduced tumor formation and tumor size. This effect was attributed to the induction of apoptosis and disruption of cell cycle by UA [125]. A 1% UA supplemented diet was found to also be effective for preventing prostate cancer metastasis in TRAMP mice [126]. In this study, UA was found to downregulate CXCR4 in prostate cancer cells, which correlated with an inhibition of CXCL12-induced migration, reducing metastasis of prostate cancer cells. In a follow-up study, UA supplementation TRAMP mice diets for 12 weeks exhibited delayed tumor formation and reduced tumor growth and increased survival [127]. These authors reported that UA decreases activation of NK-κB, STAT3, AKT, and IKKα/β phosphorylation in prostate tissues, resulting in decreased TNF-α and IL-6 levels.

## 6. Neurological Disorders and Other Diseases of the Brain

Neurons are susceptible to oxidative damage which is thought to be the underlying cause of many neurodegenerative diseases. UA’s role has been investigated in preventing neurodegeneration through the reduction in ROS production and inflammation through the upregulation of antioxidant enzymes and the downregulation of inflammatory pathways (Table 1). In various rodent models of neurotoxicity, UA protected against oxidative stress and free radicals in various regions of the brain [69,70,71,74,128,129]. Specifically, UA administered through oral gavage protected senescent mice from D-galactose-induced neurotoxicity by increasing the activity of antioxidant enzymes (SOD, catalase (CAT), glutathione peroxidase (GPx) and glutathione reductase (GR), reducing general lipid peroxidation in the brain [70], and by decreasing advanced glycation end products (AGEs), ROS, and protein carbonyl levels, mainly by down-regulating iNOS, COX-2, and various inflammatory cytokines mediated through NFκB [71]. Furthermore, UA protects the brain from ischemic injury in mice through activation of the NRF2 pathway, a cellular antioxidant response system [76]. The neuroprotective effects of UA, therefore, appear largely due to its potent anti-inflammatory properties. Mice fed an HFD that received a daily oral gavage of UA (10 mg/kg/day) showed reduced cognitive impairments, effects that appear to be mediated by inhibiting endoplasmic reticulum (ER) stress and the NFκB signaling pathway, and restoring insulin signaling and the mammalian target of rapamycin (mTOR) pathway [73]. Furthermore, in a model of lipopolysaccharide (LPS)-induced brain inflammation, UA administered by oral gavage (10 or 20 mg/kg/day) significantly improved cognitive deficits, which was attributed to decreased inflammatory mediators, including COX2, iNOS, TNFα, and various NFκB-dependent inflammatory interleukins [72]. Tsai and Yin found UA and OA protected against hydrogen peroxide (H_2_O_2_) or 1-methyl-4-phenylpyridinium ion (MPP^+^)-induced neuronal cell damage in a concentration-dependent manner [28]. Interestingly, UA was more potent than OA in protecting the PC12 cells from plasma membrane damage and preventing the release of inflammatory mediators, IL-6 and TNF-α.

Of note, UA has been proposed as a potential therapeutic for Alzheimer’s disease due to UA’s ability to reduce amyloid β binding to CD36, an important step in microglial activation and the onset of neuroinflammation [74]. Furthermore, UA was found to attenuate early brain injury after subarachnoid hemorrhage and shows promise as a neuroprotective compound for the treatment of Parkinson’s disease [75,77]. Together, these findings highlight UA’s therapeutic potential in neurological disorders and neurodegenerative diseases.

## 7. Biological Effects of Naturally Occurring Analogues of UA

In addition to UA, many of its naturally occurring analogues have also been investigated for their biological effects and potential health benefits. Interestingly, many of the analogues are found in olive oil, which is thought to be responsible for many of the beneficial health effects of the Mediterranean diet, suggesting a possible additive or even synergistic effect of these compounds in human health. Based on the reported health benefits and potential relevance for human disease prevention, we will limit our discussion to the following three natural UA analogues: asiatic acid (AA), corosolic acid (CA), and 23-hydroxy UA (23-OH UA, Table 3).

### 7.1. Asiatic Acid

Asiatic acid (AA) has also been investigated as an anti-cancer compound. Studies in breast cancer cells [130], human melanoma cells [131], and human hepatoma cells [132] demonstrate that AA induces apoptosis in cancer cells (Table 3). The metabolic effects of AA have also been examined. Yan et al. supplemented HFD-fed mice diets with 10 or 20 mg/kg/day of AA and found it increases insulin sensitivity and protects the mice from hepatic steatosis. ROS production, hepatic lipid accumulation, IL-1β TNFα and IL-6 secretion were also suppressed in HFD-fed mice that received the higher AA dose [133]. Ramachandran et al. also demonstrated that AA reversed streptozotocin-induced diabetes in rats that received AA orally for 45-days before streptozotocin injection [134]. AA has also been shown to improve insulin sensitivity in Sprague-Dawley rats with metabolic syndrome [135]. The authors found that a dose of 20 mg/kg of AA was effective at reversing the high-carbohydrate, high-fructose diet-induced insulin resistance, hypertension, and inflammation. Importantly, they found AA supplementation also restored eNOS/iNOS expression to normal levels in these rats.

### 7.2. Corosolic Acid

Corosolic acid (CA) has also been demonstrated to have protective effects against metabolic syndrome in rats [136] (Table 3). Yamada et al. investigated CA’s anti-diabetic mechanisms and found it inhibited gluconeogenesis in rat liver by increasing fructose 2,6-bisphosphate and decreased cAMP levels, inhibiting PKA activity and by increasing glycolysis [137]. Another possible mechanism for CA’s anti-diabetic effect may be its ability to promote GLUT4 translocation. Hind limb skeletal muscle of diabetic KK-Ay mice that were orally administered 10 mg/kg of CA showed higher levels of GLUT4 translocation than those in control mice [138]. These findings were replicated by Shi et al. in CHO/hIR cells [139]. These authors reported that CA inhibits protein activity of negative regulators of insulin uptake; tyrosine phosphatase1B and src homology phosphatase-1 and 2 activities.

### 7.3. 23-Hydroxy Ursolic Acid

Based on structure-function studies using ursolic acid and nine of its naturally occurring UA analogues, Nguyen et al. identified 23-hydroxy ursolic acid (23-OH UA) as a novel, naturally occurring triterpenoid with potential health benefits [19] (Table 3). 23-OH UA is a phytochemical found in the leaves of Lagerstroemia speciosa or giant crepe-myrtle native to South East Asia, and leaves and twigs of Juglans sinensis, a walnut tree found in East Asia [141]. Like UA, 23-OH UA prevented nutrient-stress induced dysfunction in THP-1 monocytic cells and human blood monocytes. In HFD-fed LDLR^−/−^ mice, an established mouse model of human atherosclerosis, both dietary UA and 23-OH UA supplemented at 0.05% to the HFD prevented dyslipidemia-induced loss of MKP-1 activity and hyper-chemotactic activity, hallmarks of blood monocyte dysfunction, without affecting plasma lipids or blood glucose levels or white blood cell and monocyte counts. Despite their similar mechanism of action, dietary 23-OH UA was significantly more effective in preventing atherosclerotic lesion formation and weight gain than UA. In a follow-up study, the same group confirmed the potent anti-obesogenic properties of 23-OH UA in a mouse model of diet-induced obesity and reported that 23-OH UA also improves glucose tolerance, prevents hyperleptinemia, preserves blood monocyte function, and reduces the recruitment of monocyte-derived macrophages into adipose tissues during nutrient stress. The authors provide evidence that the mechanism of action of 23-OH UA appears to involve the conversion of macrophages into anti-inflammatory, potentially inflammation-resolving phenotypes, which appears to contribute to the reduced adipose tissue inflammation seen in 23-OH UA-supplemented mice. Together these data suggest that 23-OH UA may serve as an oral therapy for patients at risk for obesity, impaired glucose tolerance, and cardiovascular diseases.

### 7.4. Other Ursolic Acid Analogues and Related Pentacyclic Triterpenoids

Other less studied naturally occurring analogues of UA include erythrodiol (ED), hederagenin (HG), and madecassic acid (MA). They have all been investigated as anti-cancer agents. ED, another analogue found in olive oil, was also found to have anti-cancer properties. ED inhibited proliferation and promoted apoptosis in HT-29 adenocarcinoma cells [142]. Adding ED (10 mg/kg) to a western diet after 12 weeks significantly decreased lipid droplets in liver male ApoE/ApoA1-deficient mice [143]. Hepatic transcriptome analysis of these mice revealed altered gene expression in pathways related to detoxification, protein metabolism, and nucleic acid-related metabolites. ED also stabilized ABCA1, a key transporter for cholesterol efflux, in THP-1-derived human macrophages [144]. Yu et al. reported that intraperitoneal injection of HG attenuated cerebral ischemia/reperfusion-induced apoptosis and inflammatory cytokine expression and reduced cerebral infarction via the MLK3 signaling pathway [145]. HG also reduced bleomycin-induced pulmonary fibrosis in rats by decreasing the levels of α-SMA, collagen I, hydroxyproline, and decreased inflammatory cytokines (TNF-α and IL-6) as well as phosphorylation of JNK and NFAT4 in a dose-dependent manner [146]. MA showed anti-diabetic properties. Daily i.p. injections of MA (20 mg/kg, daily) in 5-week-old male C57BL6J mice fed a HFD for 4 weeks, significantly improved systemic insulin sensitivity [147]. MA also restored vascular relaxation and increased NO bioavailability in these mice through AKT and eNOS phosphorylation. Interestingly, in our studies, neither ED nor MA showed protective effects in nutrient stress-induced monocyte dysfunction whereas HG was nearly as potent in preventing monocyte dysfunction as UA and 23-OH UA [19], suggesting a common molecular target for UA, 23-OH UA and HG.

UA’s structural isomer OA, an oleane-type triterpenoid (Figure 1), was traditionally used in folk medicine as an anti-inflammatory and is currently marketed in China for use against liver diseases [148,149] (Table 3). OA’s pharmacokinetics has been described in rodents as well as humans. The pharmacokinetics of OA in rats was described by Jeong et al. [150]. They orally administered OA at 10, 25, and 50 mg/kg intravenously and found absolute oral bioavailability to be 0.7% at 25 and 50 mg/kg. The low bioavailability may be a result of poor absorption or fast clearance. Song et al. gave 40 mg of OA in capsule form to 18 healthy men and used HPLC tandem MS to determine levels of OA in human plasma. They found the highest concentration of OA in the plasma level to be 12.12 ng/mL (0.03 μM) at 5.2 h [150].

Like UA, OA has been reported to have anti-inflammatory properties such as ameliorating formaldehyde-induced arthritis in rats [151]. These authors also found the median lethal dose for OA to be greater than 2 g/kg, suggesting low toxicity in rodents. OA is also known to have anti-viral properties [152,153]. Human peripheral blood mononuclear cells (hPBMC) isolated from healthy donors and HIV-infected donors were treated with varying doses of OA (10–80 μM). At 80 μM, the authors found 60% inhibition of HIV replication and 90% inhibition of HIV-1 protease, an essential protein for HIV replication [152]. However, the concentrations used in this study should be considered supra-physiological. Kashiwada et al. also found OA to have anti-HIV properties [153]. OA-treated H9 cells showed reduced replication of HIV-1 (EC_50_ = 1.7 μg/mL or 3.7 μM). OA has also been reported to have anti-cancer effects. In a cell culture study with four different liver cancer cell lines, Yan et al. found OA, along with UA, to decrease cell viability, increase DNA fragmentation, and increase caspase-3 and caspase-8 levels, indicating their potential as anti-cancer agents [93]. In addition to liver cancer, OA has proven to be effective against lung cancer cell lines [154], leukemia cells [155], and is currently being used in phase 1 clinical trials as an anti-cancer therapeutic [156].

Betulin is a plant-derived pentacyclic triterpene metabolite of the lupine type (Figure 1) and found in large quantities in the outer bark of birch trees [157]. Betulin shows a wide range of pharmaceutical effects such as anti-HIV, anti-inflammatory, and anti-cancer properties [146,158]. Kamaraj et al. reported that ovalbumin (OVA)-induced lung inflammation and hypersensitivity were attenuated by reducing the production of ROS and pro-inflammatory cytokines through the down-regulation of MMP-9 expression, tissue transglutaminase (tTG), TGF-β1 gene expression and by reducing TREM-1, p-IκB, and NF-κB p65 protein levels in the lung [159]. In an experimental mammary cancer model, orally supplemented betulin restored antioxidant activity and modulated the expression of both MAPKs and AhR/Nrf2-associated proteins [160], indicating that betulin has strong anti-inflammatory and anti-cancerogenic properties.

Several synthetic triterpenoids have also been shown to protect cancer cells, macrophages, and neutrophils from oxidative stress, inflammatory stimuli, and cell death [156,161,162]. Of these, the synthetic oleanolic acid-based CDDO series is best characterized. Thimmulappa et al. found that CDDO upregulated the Nrf2 pathway, an important antioxidant pathway critical for activating phase 2 genes [161] (Table 3). Phase 2 genes are upregulated during oxidative stress, members include heme oxygenase 1 (HO-1), glutathione S-transferase (GST), and NADP-quinone reductase and therefore have been explored as possible cancer therapeutic targets [163,164,165]. CDDO has also been demonstrated to increase HO-1 and GST and reduce LPS-induced inflammation as well [162]. CDDO is also a partial agonist of PPARγ [166]. Mice with breast cancer saw a reduction in tumor sizes when their diets were supplemented with 40 mg/kg of CDDO [165] and CDDO was shown to be effective against triple-negative breast cancer by targeting tumor stem cells [164].

## 8. Conclusions

The data reviewed here reveal that UA has a wide range of biological activities and is able to both prevent and treat a variety of pathologies in animal models of human diseases, ranging from metabolic disorders and chronic inflammatory diseases to cancer and neurological diseases. Whether these promising results will translate into real health benefits in humans remains to be explored as do UA’s bioavailability, pharmacokinetics, efficacy, and safety for humans. Furthermore, pharmaceutical efforts should focus on identifying the molecular target(s) of UA and on developing UA derivatives with better bioavailability. We hope this review will stimulate more research into UA and its analogues and derivatives and will ultimately lead to more human trials with this exciting and promising compound.

## Figures and Tables

**Table 1:** UA in animal disease models.

Disease	UA	Delivery	Model	Outcome	References
CVD	60 mg/kg body weight	IP	Dahl salt-sensitive rat model	↓ HTN, BG, and TC; ↑ GPx and SOD	[20]
CVD	85 mg/kg body weight	subq IP	Windsor rats	against ISO-induced MI, ↓ CK-MB, LDH, LDL, TG, and FFA	[57]
CVD	85 mg/kg body weight	subq IP	Windsor rats	↑ Bcl-2, Bcl-xl and ↓ of Bax, caspase-3, -8, and -9, cytochrome c, TNF-alpha, and FAS. ↓ lipid peroxidation markers and ↑ antioxidant enzymes and non-antioxidant enzymes in the plasma and heart tissue of ISO-induced MI	[58]
CVD	50 mg/kg	oral gavage	STZ-Diabetic mice	↓ aortic damage, RAGE, P22, and NFĸB	[59]
CVD	0.05%	HFD	LDLR-KO mice	↓atherosclerotic plaque size and weight gain	[19]
CVD	0.20%	HFD	STZ-treated LDLR-KO, mice	↓ atherosclerosis lesion formation, fewer infiltrating macrophages, ↓ BG, Alb/Crt ratio, inflammatory blood monocytes, ↑ low inflammatory blood monocytes	[60]
Diabetes	0.05%	HFD	STZ-Diabetic mice	Protects pancreatic islet cells and ↑ insulin secretion	[61]
Diabetes	5 mg/kg	HFD	C57BL/6J mice	UA combined with rosiglitazone ↓ whole BW gain, and can have profound responses to rosiglitazone or metformin.	[62]
Diabetes	0.01% and 0.05%	AIN-76 semisynthetic diet	STZ/NA-Diabetic mice	Significant improved diabetic outcomes and stimulated T-lymphocytes in the thymus	[52]
Diabetes	5 mg/kg	HFD	C57BL/6J mice	UA combined with rosiglitazone ↓ hepatic marker enzyme activities and ↓ lipid accumulation in liver	[63]
Kidney Disease	0.01%	Standard rat chow	STZ-Diabetic mice	↓ glomerular hypertrophy, collagen accumulation, and suppressed activation of STAT-3, ERK1/2, JNK and iNOS overexpression	[64]
Kidney Disease	0.05%, 0.1% and 0.2%	64 g starch, 23 g protein, 3.5 g fat, 5 g fiber, 1 vitamin mixture and 3 salt mixtures	STZ-Diabetic mice	↑ kidney function ↓ flux through the renal polyol pathway, and ↓ AGEs formation in urine	[65]
Kidney Disease	0.2%	Standard rat chow	STZ-Diabetic mice	↓ UAE, renal oxidative stress, NF-KB activity, and P-selection expression	[66]
Kidney Disease	25 and 50 mg/kg	oral gavage	ICR mice	UA prevents CCl4-induced nephrotoxicity, ROS, DNA damage, and proinflammatory markers	[37]
Kidney Disease	2, 5, and 10 mg/kg	orally	Wistar albino rats	UA protected kidneys from gentamicin-induced damage	[67]
Kidney Disease	0.2% in diet	Standard rat chow	Wistar rats	↓ UAE, renal oxidative stress level, NF-κB activity, and P-selectin expression.	[42]
Liver Disease	1–100 µM	Incubation medium	Human liver microsomes	UA regulation of cytochrome P450 shows hepatoprotective properties	[44]
Liver Disease	50 mg/kg	IP	Wistar rats	Induced apoptosis in liver-damaging hepatic stellate cells while maintaining normal hepatocyte function	[45]
Liver Disease	50 mg/kg	oral gavage	C57/BL6 WT mice	↓ oxidative stress through activation of LKB1-AMPK signaling	[46]
Liver Disease	25 and 50 mg/kg	intragastrically	ICR mice	↓ CCl(4)-induced lipid peroxidation levels and depleted TAC levels in liver. ↓ CYP2E1, TNF-α, IL-1β and COX-2, JNK, p38 MAPK, ERK, and inactivation of NF-κB	[37]
Liver Disease	1, 10, and 100 µg/mL	cell culture UA treatment	Albino Druckery rats	UA isolated from Eucalyptus tereticornis improved liver function measured by AST, ALT, and ↑ glutathione, α-tocopherol, and ascorbic acid	[38]
Liver Disease	10, 20, and 40 mg/kg/day	intragastrically	Wistar albino rats	Pure UA improved liver function measured by AST, ALT, and ↑ glutathione, α-tocopherol, and ascorbic acid	[39]
Liver Disease	0.125%, 0.25%, and 0.50%	HFD	Sprague-Dawley rats	Significantly reversed HFD-induced hepatic steatosis and liver injury	[43]
Liver Disease	5, 20, and 80 µM	cell culture UA treatment	Cultured HepG2 cells	↑ PPARα binding to its response element but did not directly bind PPARα in the liver hepatocyte cell line, HepG2 cells	[47]
Liver Disease	HepG2 (6.25, 12.5, and 25 µM) and HUVECs (5, 10, 20 µM)	cell culture UA treatment	HUVECs and HepG2 cells	↓ inflammatory cytokine production induced by IL-6 in HepG2 cells	[48]
Liver Disease	0.1 and 0.05%	AIN-76 semisynthetic diet	STZ/NA-Diabetic mice	↓ FBG, TG, FFA, TC and VLDL, LDL. ↓ hepatic G6-P activity and ↑ glucokinase activity, the glucokinase/G6-P ratio, GLUT2 mRNA levels and glycogen content. ↑ aldose reductase activity, ↓ SDH	[52]
Metabolic Disease	50 µM	cell culture UA treatment	C2C12 cells	Inhibited mTORC activation by leucine through suppression of mTOR lysosomal localization	[55]
Metabolic Disease	250 mg/kg	IP	Sprague-Dawley rats	UA sustained exercise-induced mTORC1 activity	[56]
Metabolic Disease	40 mg/kg body weight	IP	C57Bl/6 mice	↑ muscle mass by inhibiting skeletal muscle atrophy and improved metabolic outcomes	[50]
Metabolic Disease	0.5 g/kg	HFD	STZ-Diabetic mice	↓ blood glucose, TC, FFA, TG, and improved liver function	[41]
Metabolic Disease	125 nM, 250 nM, 500 nM and 1 µM	cell culture UA treatment	CHO/hIR cells	Inhibition of PTP1B ↓ blood glucose. PTP1B is a phosphatase inhibitor of insulin-mediated signaling.	[68]
Neuro. Disease	5, 10, and 15 µM	cell culture UA treatment	Sprague-Dawley rats	↓ free radical generation in primary rat hippocampus neurons in response to kainite	[69]
Neuro. Disease	10 mg/kg/day	oral gavage	Kunming strain mice	↑ activity of antioxidant enzymes, SOD, CAT, GPx, and GR and ↓ general lipid peroxidation in the brain	[70]
Neuro. Disease	10 mg/kg/day	oral gavage	Kunming strain mice	↓ AGEs, ROS, PCO levels, and down-regulated iNOS, COX-2, and various inflammatory cytokines mediated through NFκB, all found in the prefrontal cortex of the brain	[71]
Neuro. Disease	10 or 20 mg/kg/day	oral gavage	C57BL/6J	improved cognitive deficits attributed to ↓ COX2, iNOS, TNFα and various inflammatory interleukins mediated through p38/NFκB signaling pathways	[72]
Neuro. Disease	10 mg/kg/day	oral gavage	C57BL/6J	improves cognitive impairments by inhibiting ER stress and NFκB signaling pathway, restoring insulin signaling and the mTOR pathway	[73]
Neuro. Disease	50 or 100 µM	cell culture UA treatment	CHO-CD36 and primary microglia cells	Potential treatment for Alzheimer’s Disease due to ↓ amyloid β binding to CD36	[74]
Neuro. Disease	25 and 50 mg/kg	IP	SD rats	↓ oxidative stress attenuating EBI after SAH	[75]
Neuro. Disease	130 mg/kg	IP	Nrf2−/− and WT rats	Protects brain from ischemic injury through activation of NRF2 pathway	[76]
Neuro. Disease	100 nM	cell culture UA treatment	Patients with parkin or LRRK2 mutations	↑ activity of the mitochondrial respiratory chain and displayed drug-like dose-response curves for Parkinson’s Disease	[77]
Obesity, Diabetes	0.05%	HFD	C57Bl/6 mice	improved glucose tolerance and wt maintenance while ↓ lipid accumulation in liver	[42]
Obesity	0.05%	Drinking Water	C57Bl/6 mice	↓ visceral adiposity, total BW, BG, and lipid	[49]
Obesity	0.14% and 0.27%	HFD	C57Bl/6 mice	↑ muscle mass, skeletal muscle glucose uptake, and BAT resulting in ↓ obesity, hepatic steatosis, and improved glucose tolerance	[40]
Obesity	2.5 to 10 µM	cell culture UA treatment	3T3-L1 mouse embryo fibroblasts	Attenuated adipogenesis through the LKB1/AMPK pathway	[53]
Obesity	25, 50, and 100 µM	cell culture UA treatment	Sprague-Dawley rats	Anti-obesity mechanism by stimulating lipolysis by upregulation of ATGL in primary rat adipocytes	[54]
Obesity	Cynomorri extract, 100–360 mg/kg body weight	HFD	C57Bl/6 mice	↓ wgt gain likely to ↑ energy expenditure based on observed mitochondrial uncoupling in skeletal muscle	[51]

**Table 2:** Anti-cancer effects of UA.

	Pathway	Cancer Type	References
Induction of apoptosis	FoxM1 ↓	breast cancer cells	[97]
	Caspase ↑	melanoma cell	[98]
		endometrial cancer cell	[99]
		prostate cancer cells	[100]
		non-small cell lung cancer	[101]
		gastric cancer cell	[102].
		colon cancer cells	[103]
		bladder cancer cells	[104]
	Trail-mediated	prostate cancer cells	[105]
	COX-2 ↓	colon cancer cells	[103,106]
		gastric cancer cell	[107]
	NF-κB ↓	bladder cancer cells	[104]
		pancreatic cancer cells	[108].
		prostate cancer cells	[109]
		hepatocellular carcinoma cells	[110]
	JNK ↑	colon cancer cells	[111]
		pancreatic cancer cells	[108]
		prostate cancer cells	[112]
Inhibition of cell proliferation	MAPK ↓	endometrial cancer	[113]
		colon cancer cells	[103,114]
	STAT3 ↓	prostate cancer cells	[109]
		multiple myeloma cells	[95]
		colorectal cancer cells	[115,116]
	p53 and p21^WAF1^ ↑	non-small cell lung cancer	[117]
Inhibition of metastasis	HIF-1α ↓	neuroblastoma cells	[118]
	VEGF ↓	lung cancer cells	[119]
		colorectal cancer cells	[120]
		liver cancer cells	[93]
		neuroblastoma cells	[118]
	MMP-9 ↓	glioma cells	[94]
		liver cancer cells	[93]
		lung cancer cells	[119]
	ICAM-1 ↓	liver cancer cells	[93]
		lung cancer cells	[119]

**Table 3:** Effects of natural UA analogues.

Analog	Dose	Delivery	Model	Outcome	References
Asiatic acid	2.5, 5, 10 and 20 µM	cell culture AA treatment	Breast cancer cell lines MCF-7 and MDA-MB-231	Cell growth inhibition by inducing cancer cells to undergo S-G2/M phase arrest and apoptosis	[130]
Asiatic acid	10, 20, 30, 40 and 50 µM	cell culture AA treatment	SK-MEL-2 human melanoma cell line	↓ cell viability, induced apoptosis in SK-MEL-2 cells, ↑ ROS, enhanced Bax expression, and induced caspase-3 activity	[131]
Asiatic acid	10, 20, 30, 40, 70 and 100 µM	cell culture AA treatment	HepG2 human hepatoblastoma cell line	↓ cell viability, induced apoptosis in HepG2 human hepatoma cells, ↑ intracellular Ca2+ level and p53 expression	[132]
Asiatic acid	10 or 20 mg/kg/day	oral gavage	C57BL/6 mice	↑ insulin sensitivity, protected mice from hepatosteatosis, ↓ ROS production, hepatic lipid accumulation, and IL-13B secretion with high AA dose	[133]
Asiatic acid	5, 10 and 20 mg/kg BW	oral	STZ-diabetic mice	Reversed STZ-induced diabetes, potentially regulates CHO metabolism by modulating diabetic-regulatory enzymes	[134]
Asiatic acid	10 or 20 mg/kg/day	intragastrically	Sprague-Dawley rats	Improved HCHF diet-induced insulin sensitivity, lipid profiles, hemodynamic parameters, oxidative stress markers, plasma TNF-α, NOx, and recovered abnormality of eNOS/iNOS expressions	[135]
Corosolic acid	0.072%	HFD	SHR-cp rats	↓ blood pressure, serum FFAs, oxidative stress markers, myeloperoxidase markers, and high sensitivity C-reactive protein	[136]
Corosolic acid	20–100 µM	Syringe pump infused	Wistar rats	Inhibited gluconeogenesis in liver by ↑ Fru-2,6-BP, ↓ cAMP levels, inhibiting PKA activity and ↑ glycolysis	[137]
Corosolic acid	10 mg/kg BW	oral	KK-Ay mice	Hypoglycemic effect derived from ↑ GLUT4 translocation in muscle	[138]
Corosolic acid	250 and 500 nM	cell culture CA treatment	CHO/hIR and L6 myoblast cells	Enhanced glucose uptake by ↑ GLUT4 translocation mediated by insulin pathway activation, inhibited PTP1B, T-cell-PTP, src phosphatase 1 and 2 activity	[139]
23-Hydroxy Ursolic Acid	0.05%	HFD	LDLR-KO mice	↓ atherosclerotic plaque size and weight gain, more potent than ursolic acid	[19]
23-Hydroxy Ursolic Acid	0.2%	HFD	C57BL/6 mice	↑ glucose tolerance, ↓ weight gain, hyperleptinemia, macrophage recruitment, and adipose tissue inflammation	[140]

## Data Availability

The data is contained within the article.
